# Measurement Methods for Fiber Volume Fraction of Fiber-Reinforced Polymer Composites

**DOI:** 10.3390/polym18040434

**Published:** 2026-02-09

**Authors:** Xudan Yao, Yaxin Wang, Haolin Wang, Aiwei Zhan, Yichen Wu, Yaqi Wang, Wandong Wang

**Affiliations:** 1 School of Aeronautics, Northwestern Polytechnical University, Xi’an 710072, China; 2 National Key Laboratory of Strength and Structural Integrity, Xi’an 710072, China

**Keywords:** fibre volume fraction (FVF), fibre-reinforced polymer (FRP) composites, measurement methods

## Abstract

Fiber-reinforced composites are extensively utilized in mass-critical structures spanning aerospace, automotive, civil, etc., owing to their exceptional specific strength and stiffness. Fiber volume fraction (FVF) is a critical parameter for evaluating the performance of the composites. As a consequence, different measurement methods have been developed in recent decades, including resin removal method, thickness measurement method, microscopic method, etc. This paper reviews both traditional destructive (acid digestion, combustion, image analysis, etc.) and newly developed non-destructive techniques (X-ray CT, thermography, ultrasonic, XRD, eddy current, etc.), with a focus on their applicability to specific materials, measurement accuracy, operational complexity and cost. Moreover, key challenges and future directions are discussed, emphasizing the need for non-destructive testing, cost and energy efficiency, intelligent measurement and sustainability.

## 1. Introduction

Fiber-reinforced composites, particularly fiber-reinforced polymer (FRP) composites, comprising fiber and polymer matrix, exhibit superior properties, including low density, high specific strength and stiffness, corrosion resistance, and design flexibility. These advantages accelerate the application of FRPs in aerospace, automotive, railway, bridge and wind turbine blades, etc., to replace traditional materials. Mechanical properties of composites are primarily governed by volume fractions of fiber and matrix, as well as their interfacial properties. The matrix distributes the load onto and between the fibers, while the fibers are the primary load-carrying components [1]. The properties can be predicted based on the rule of mixture principles, and thus precise quantification of constituent volume fractions is important.

Different manufacturing methods have been developed and they contribute to different fiber volume fractions (FVFs), which significantly affect the mechanical and functional performance of the composites [2,3]. For instance, when the FVF of the filament wound FRP composites rises from 50% to 65%, the strength of composites improved by over 10% [4]. As a consequence, accurate determination of the FVF is essential to ensure product quality and optimize the properties of composites [3].

Traditional techniques have been widely developed and utilized, including resin removal method via combustion or chemical digestion, thickness measurement, and microscopic method using the image analyzer. Hence, corresponding standards, i.e., ISO 14127 [5] and ASTM D3171 [6], have been proposed. In the recent decades, non-destructive testing (NDT) methods, such as eddy current method, ultrasonic testing, X-ray computed tomography (CT) for three-dimensional (3D) characterization, etc., have been largely developed for detecting defects and evaluating the structural integrity of composites utilized in different fields. Among them, some methods are also applicable for measuring the fiber volume fraction of composites [7].

**Figure 1:**
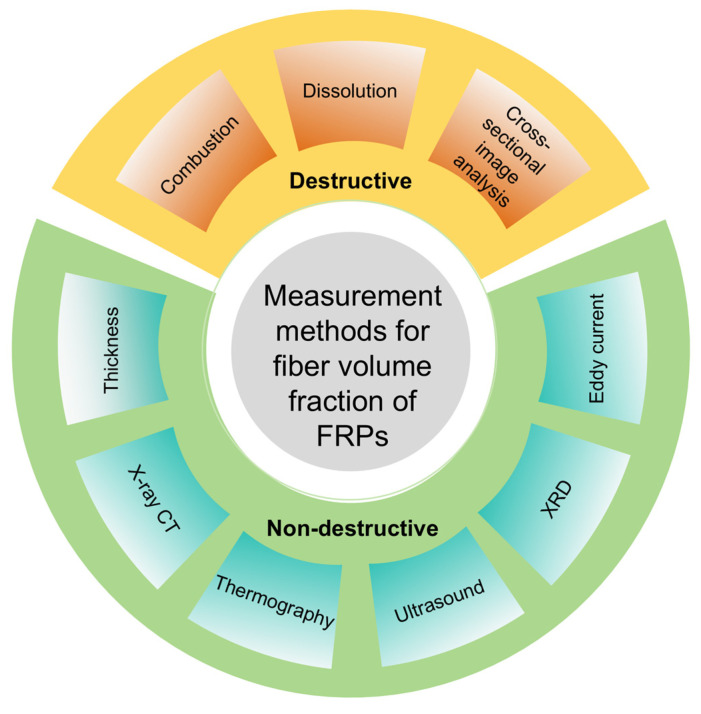
Overview of fiber volume fraction measurement methods.

This review aims to provide a comprehensive analysis of measurement methods for fiber volume fraction of fiber-reinforced composites, as shown in Figure 1, identify the scope of applications for each method, and propose perspectives on the sustainable and economically efficient strategies in the future.

## 2. Traditional Destructive Methods

Destructive methods like acid digestion or combustion were utilized in the early time and considered to be reliable and accurate. Both of them take localized samples and remove the matrix with hazardous chemicals involved in the measurement procedure. By quantifying the mass differential before (M_c_) and after (M_f_) the matrix removal, the fibre weight (W_f_) and volume (V_f_) fractions are determined by the following equations.

(1) $$W_{f}=\frac{M_{f}}{M_{c}}$$

(2) $$V_{f}=\frac{M_{f}}{M_{c}}\times \frac{\rho_{c}}{\rho_{f}}$$

Similarly, the matrix weight (W_m_) and volume (V_m_) fractions can be calculated by Equations (3) and (4).

(3) $$W_{m}=\frac{{M_{c}-M}_{f}}{M_{c}}$$

(4) $$V_{m}=\frac{M_{c}-M_{f}}{M_{c}}\times \frac{\rho_{c}}{\rho_{m}}$$

where c, f, m and v represent the composite, fiber, matrix and void. Afterwards, the void volume fraction can be obtained:

(5) $$V_{V}=1-\left(V_{f}+V_{m}\right)$$

### 2.1. Combustion

The combustion or burn-off method relies on the different thermal degradation temperatures of the matrix and reinforcements, which demonstrates particular efficacy for analyzing composites reinforced with high-temperature-resistant fibers, such as glass, quartz, or silica fibers, while organic matrices fully decompose. Its fundamental principle involves heating the composite to a temperature at which the matrix material completely combusts or decomposes, while the fibers either do not burn or experience only minimal mass loss. In addition, under conditions involving matrix retention, the carbonization ratio of the neat resin should be considered for correction during the fiber volume fraction calculation [6].

**Figure 2:**
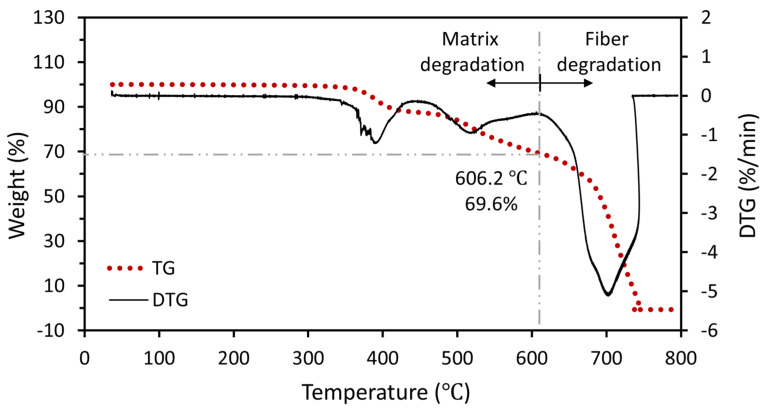
Typical TG and DTG curves of CFRP composites, with the matrix and fiber degradation regions separated and the fiber weight fraction obtained. Adapted from Ref. [8].

Apart from combustion in a furnace, thermogravimetric analysis (TGA) has been widely used for determining the fiber volume fraction (V_f_), from which W*_f_* could be obtained directly from the DTG curve, as illustrated in Figure 2, owing to its automation and continuously monitoring during the whole procedure [8,9,10]. In particular, to distinguish between char degradation of the polymer matrix and carbon fiber degradation, Grund et al. [3] conducted a systematic investigation on the effects of heating rate, analysis of atmospheric and sample size, and proposed an optimal mass-rate-controlled heating program.

As an intrinsically destructive testing protocol, this method completely removes the matrix material, rendering the tested sample unusable afterwards. Additionally, the process may emit harmful gases (e.g., organic compounds and potential toxic byproducts), requiring proper laboratory conditions equipped with fume extraction systems or ventilation facilities to ensure operational safety.

Testing duration varies with specimen geometry (notably thickness), matrix composition, and heating apparatus specifications. Complete thermal processing, including temperature ramp, isothermal combustion, and controlled cooling, typically requires 1–2 h per sample. Compared to other methods, the combustion method offers distinct advantages in cost efficiency, requiring only basic laboratory equipment, i.e., a furnace, compared to other methods.

### 2.2. Dissolution

The dissolution or digestion method employs selective chemicals to dissolve the matrix of composites, including polymer and metal matrices, which has been widely utilized [11,12,13]. For example, concentrated nitric acid can be used to digest epoxy resin, steel, copper, etc., hydrochloric acid for steel, titanium, copper, aluminum, etc., sodium hydroxide solution can dissolve aluminum, brass, etc., aqueous mixture of sulfuric acid and hydrogen peroxide for epoxy, phenolic, polyamide and thermoplastic resin, etc., and elevated temperatures are needed for most circumstances [6].

Hassan et al. [14] investigated the influence of the acid volume, digestion duration, and temperature on the resin extraction and found that the temperature critically contributed to the testing procedures of the digestion process and the results attained. In addition, a mathematical model was proposed as well as optimum parametric conditions for particular acid digestion tests.

Similar to combustion, this method is also inherently destructive and therefore unsuitable for components requiring structural integrity preservation. Leveraging its economic advantages, the dissolution procedure is time-consuming, involving specimen preparation, dissolution, filtration, drying and waste disposing; it demands multi-hour commitments modulated by substrate reactivity and kinetic constraints. Despite procedural simplicity, this method presents significant health, safety and environmental hazards, and the waste must be disposed properly; it is also time-consuming and is a localized measurement.

### 2.3. Cross-Sectional Image Analysis

Apart from combustion and dissolution, cross-sectional image analysis has also been widely applied for measuring the fiber volume fraction, with the advantages of no harmful byproducts and fast evaluation. This method is well-suited for measuring composite materials with distinct optical characteristics, such as carbon fiber or glass fiber-reinforced plastics. There are two routes to achieve the FVF, i.e., the areal method and the fiber counting method. The former one is more commonly used currently, while the latter one is more accurate [15]. In addition, to improve the accuracy, the sample surface must be grinded and polished until the cross-section morphology is clearly visible under the microscope [5].

**Figure 3:**
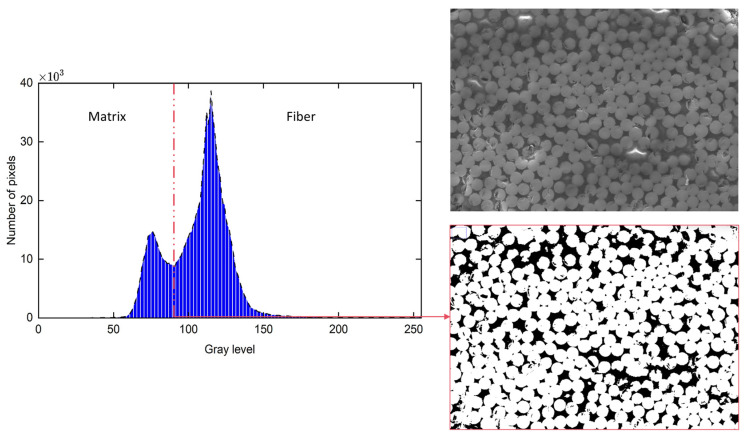
Typical cross-sectional image analysis. SEM image, histogram of gray level depicting fiber and matrix separation and image analysis for fiber volume fraction.

For unidirectional composite laminates, specimens shall be cut off perpendicular to the axial direction of the fiber. For orthogonal and multidirectional composites, specimens shall be cut off along each cross-section perpendicular to every axial direction of the fiber. The fiber volume fraction (V_f_) can be calculated from the following equations.

(6) $$V_{f}=\frac{\sum_{i=1}^{n}V_{fi}}{n}$$

(7) $$V_{fi}=\frac{N_{i}\cdot \bar{A_{f}}}{A_{i}}$$

where the $V_{fi}$, n, $N_{i}$, $\bar{A_{f}}$ and $A_{i}$ represent the fibre content in each observation field, number of observation fields, number of fibers in each observation field, average cross-sectional area of the fiber and area of each observation field [5]. As shown in Figure 3, similar methods can be applied to calculate the matrix and void volume fractions, with the relationship in Equation (8).

(8) $$V_{f}+V_{m}+V_{V}=1$$

With advancements in optical technology and digital image processing, modern cross-sectional image analysis integrates high-resolution microscopy, automated image acquisition systems, and advanced data processing software. This evolution has significantly broadened its application in fiber volume fraction measurement. Crucially, this approach requires only moderately priced equipment (e.g., optical microscope), establishing it as a cost-efficient, mid-to-low-range characterization technique.

Apart from optical microscopy [16], scanning electron microscopy (SEM) images have also been applied for micrograph analysis and FVF calculation [17]. Current research primarily focuses on increasing the degree of automation in image analysis, enhancing the accuracy of image processing algorithms, and extending the methodology to 3D image analysis [18].

## 3. Non-Destructive Methods

### 3.1. Thickness Method

Fibre volume fractions ($V_{f}$) of the composites can be evaluated through calculation based on the thickness (d) of the composites, combined with known values of the density of the fibre (ρ_f_), number of plies (n) and area weight (A_w_) of the fabric or fibres in the plies of the prepreg [19,20].

(9) $$V_{f}=\frac{nA_{w}}{\rho_{f}d}$$

Notably, this method presents significant advantages in terms of cost-effectiveness and procedural simplicity. It is applicable to relatively flat panels made with reinforcement of known and consistent areal weight [6]. Meanwhile, the accuracy of the measurement will be significantly affected by the uniformity of the surface, and therefore smoothness of the composite should be considered for correction when needed.

### 3.2. X-Ray Computed Tomography

X-ray computed tomography (CT) is gaining prominence as a critical non-destructive evaluation method, particularly for phenomena where 3D structural dynamics are essential or temporal evolution of critical features warrants investigation [21,22,23,24,25,26,27,28]. The inherent heterogeneity and multiscale architecture of composites demand 3D characterization, while mapping defect nucleation and progression is vital to structural integrity assurance [29]. X-ray CT with modern scanners enables multiscale analysis spanning full-component, laminate, fiber-tow, and individual-fiber resolutions [29].

**Figure 4:**
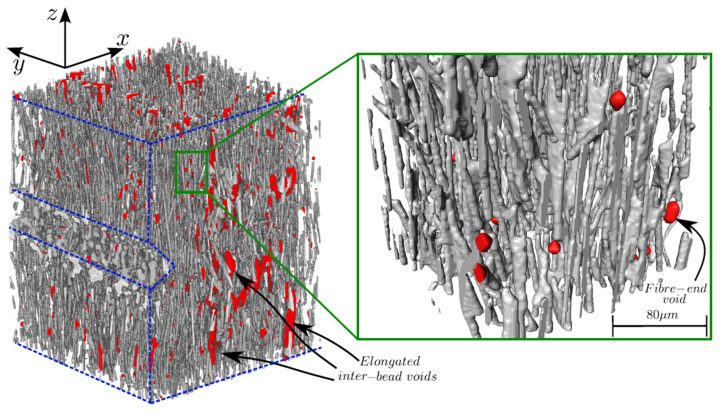
X-ray CT of CFRP composites and the 3D visualization with fibers, voids and the matrix are rendered grey, red and transparent respectively. Boundaries are highlighted in blue dashed lines, and the inlet shows a close-up view of the microstructure highlighting fiber-end spherical voids. Reprinted from Ref. [32].

X-ray CT systems can deliver high-fidelity 3D characterization of fiber distribution, manufacturing defects, and in-service damage evolution non-destructively. This capability eliminates traditional destructive cross-sectioning, which requires skilled preparation while risking sample alteration or material loss. Reconstruction of the 3D void and fiber distribution can be used for analyzing the void and fiber volume fraction [30,31]. As shown in Figure 4, after the X-ray CT scanning, the obtained projections were reconstructed and stacked to form 3D images, from which the fibers and pores could be observed [32,33]. Afterwards, image processing and segmentation will be performed; high intensity pixels corresponding to fibers can be extracted to determine the fiber volume fraction. Similarly, void volume fraction could be obtained while facing challenges in detection and segmentation due to large voids containing a combination of low and high intensity pixels [33,34]. Compared with the cross-sectional image analysis, the obtained fiber volume fractions are comparable, while void volume fractions are relatively lower by optical micrograph due to polymer filling during polishing [33].

### 3.3. Thermography

**Figure 5:**
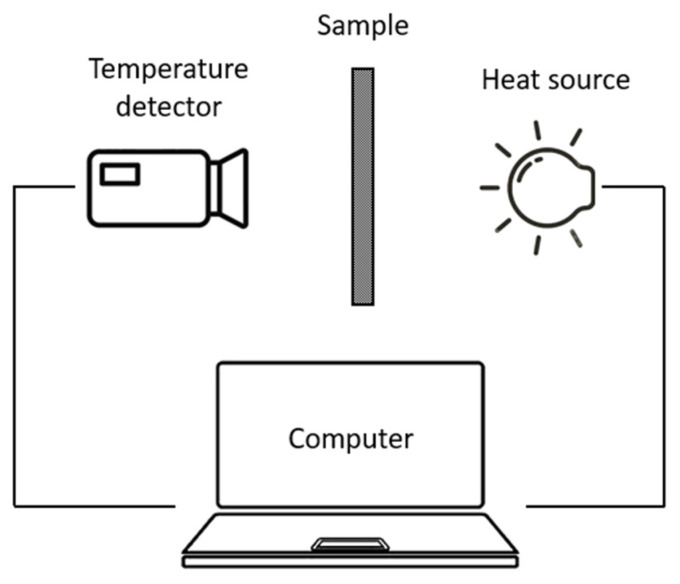
Measurement setup for the thermography method. Adapted from Ref. [37].

In 1990s, Zalameda et al. [35,36,37] reported that the FVF can be determined by measuring the thermal diffusivity (α), with porosity neglected or determined ultrasonically. The thermal diffusivity measurement setup, as shown in Figure 5, consists of the heat source, temperature detector and computer. The diffusivity can be obtained from the fitted result with the thickness known. The equivalent volumetric heat capacity can be denoted by the rule of mixture:

(10) $$({\rho c)}_{c}=V_{f}({\rho c)}_{f}+(1-V_{f}-V_{v})({\rho c)}_{m}$$

where ρ is the density, c is the specific heat, and the heat capacity of voids can be neglected.

The equivalent thermal conductivity of the composites ($k_{c}$) perpendicular to the fibres can be obtained by different models, for instance, the series model or stacked plate model (SPM):

(11) $$k_{c}=\frac{k_{f}k_{m}}{k_{m}V_{f}+k_{f}(1-V_{f})}$$

where $k_{f}$ and $k_{m}$ are the thermal conductivity of the fiber and matrix respectively.

Springer and Tsai [38] reported the square packing array model (SPAM) which takes dispersed fibers, assumed to be square filaments, into account.

(12) $$k_{c}=(1-\sqrt{V_{f}})k_{m}+\frac{k_{m}\sqrt{V_{f}}}{\left(1-\sqrt{V_{f}}\left(1-\frac{k_{m}}{k_{f}}\right)\right)}$$

In addition, Hashin and Rosen [39] derived the Composite Circular Assemblage (CCA) model, which takes into account the circular geometry of the filament.

(13) $$k_{c}=k_{m}\frac{k_{m}\left(1-V_{f}\right)+k_{f}(1+V_{f})}{k_{m}(1+V_{f})+k_{f}(1-V_{f})}$$

Combining the equivalent heat capacity and thermal conductivity, the effective one-dimensional diffusivity can be calculated as follows:

(14) $$\alpha =\frac{k_{c}}{{\left(\rho c\right)}_{c}}$$

The relationship between thermal diffusivity and fiber volume fraction is plotted and compared between different models, as well as different porosity loadings considered using the CCA model. Furthermore, the FVF values obtained from the thermography method based on the SPAM model were compared with the traditional destructive method, and resulted with comparable performance, indicating the feasibility of the method.

This method is sensitive to the thickness and has limited depth sensitivity, with limited applicability depending on materials. Also, the accuracy relies on theoretical models and still requires destructive calibrations for matrix thermal conductivity. In addition, pores working as thermal insulators, lowering the diffusivity, which could be corrected with the porosity determined ultrasonically.

### 3.4. Ultrasound

Ultrasonic technique is one of the most widely used non-destructive techniques in the aerospace industry, owing to its superior penetration capabilities in carbon fiber-reinforced plastics (CFRPs), as well as its high sensitivity to variable defects including impact damage, porosity, FVF variations, etc. [37,40]. Both ultrasonic attenuation and longitudinal/transverse wave velocity have been measured for analyzing porosity and fiber volume fractions [37,40,41,42]. Traditional ultrasonic [37] and noncontact laser-ultrasonic [40] methods have been developed.

During the traditional ultrasonic measurement, the transducer, composite, spacers and glass reflector plate were immersed in a water tank [37]. The longitudinal wave velocity was measured using a phase spectrum technique where a spectral phase difference is measured. This phase difference corresponds to a time difference or a shift in time, which can be seen by observing the glass plate reflection. The longitudinal velocity ($V_{l}$) is calculated for a wave propagating in the direction perpendicular to the fibres [37],

(15) $$V_{l}=\sqrt{\frac{C_{11}}{\rho }}$$

where the composite elastic constant $C_{11}$ is found related to FVF [43,44]:

(16) $$C_{11}=\frac{1}{2}\left[C_{11}^{m}+C_{12}^{m}+\left(C_{11}^{f}+C_{12}^{f}-C_{11}^{m}-C_{12}^{m}\right)V_{f}\right]-\frac{1}{2}\left[\frac{{\left(C_{11}^{f}+C_{12}^{f}-C_{11}^{m}-C_{12}^{m}\right)}^{2}V_{f}\left(1-V_{f}\right)}{\left(C_{11}^{f}+C_{12}^{f})\left(1-V_{f}\right)+{(C}_{11}^{m}+C_{12}^{m}\right)V_{f}+2C_{44}^{m}}\right]+C_{44}^{m}\left(\frac{1+\xi \eta V_{f}}{1-\eta V_{f}}\right)$$

where ξ is the matrix reinforcing factor, and

(17) $$\eta =\frac{C_{66}^{f}-C_{44}^{m}}{C_{66}^{f}+\xi C_{44}^{m}}$$

The differences in the transverse shear modulus between the fiber and matrix significantly affect the relationship between the longitudinal velocity and FVF.

Wu et al. [40] utilized the non-contact laser ultrasonic wave technique to analyze unidirectional composite laminates, with different modes of laser ultrasonic wave collected and analyzed along and perpendicular to the fiber direction. Longitudinal wave for porosity and the surface wave, which is sensitive to the in-plane elastic properties of both carbon fiber and matrix material, is used for fiber volume fraction. Then, the fiber volume fraction is further corrected by the laminate porosity to achieve higher accuracy. For CFRP composites, the total ultrasound velocity consists of the contribution from both the fiber and matrix,

(18) $$v_{mea}+v_{void}=V_{f}v_{fibre}+(1-V_{f})v_{resin}$$

(19) $$V_{f}=\frac{v_{mea}+v_{void}-v_{resin}}{v_{fibre}-v_{resin}}$$

where $v_{mea}$ is the measurement result, $v_{fibre}$ and $v_{resin}$ are the laser ultrasonic surface wave propagation velocity in the pure carbon fibre and resin matrix, and $v_{mea}$ is the velocity correction due to porosity [40].

### 3.5. X-Ray Diffraction (XRD)

X-ray diffraction (XRD) is a fundamental analytical technique based on the elastic scattering of X-rays by crystalline lattices, governed by Bragg’s law (nλ = 2dsinθ). It enables critical microstructural characterization of both crystalline and semi-crystalline systems. A monochromatic beam of X-rays is allowed to incident on a sample; it reflected X-rays that are detected by a detector [45]. In 1981, Prakash [46] utilized the XRD for determining the FVF of various samples, including CFRPs and GFRPs, with the burn-off method used for calibration, and correlated FVF with diffraction peak intensity in unidirectional FRPs.

For unidirectional FRP composites, an inverse linear relationship between the peak height of relative intensity of diffracted X-rays and FVF was observed. As a consequence, this method could be used to determine the FVF of unidirectional FRPs, as absorption phenomena are playing the predominant role [46,47]. However, when it comes to woven composites, the results will be scattered [46].

### 3.6. Eddy Current Testing (ECT)

Eddy current testing (ECT) is a non-destructive technique for conductive materials. The principle is that when the probe is brought near a conducting material, eddy currents are induced in the material by the magnetic field from the probe, which in turn change the frequency of the oscillator and can be converted to a voltage. As a consequence, eddy current can be used to investigate material properties that affect the eddy current path or its magnitude [48,49]. It is applicable to both metal matrix composites and CFRPs, which have been investigated for characterizing the FVF [48,49], fiber orientation [50,51], conductivity [52], in-plane and out-of-plane waviness [53,54], etc.

**Figure 6:**
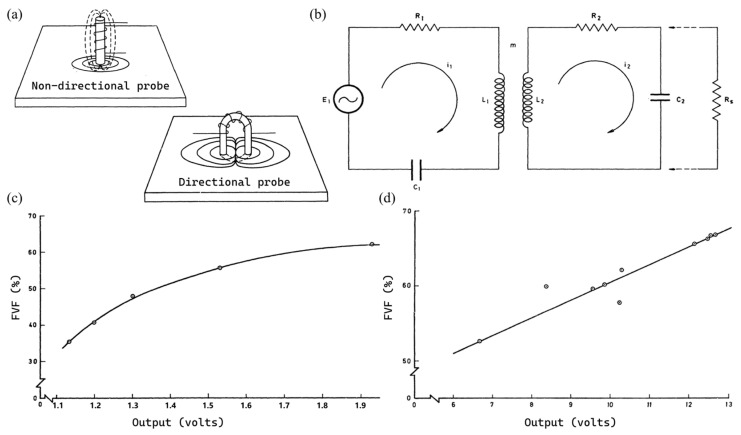
Eddy current method. (**a**) Non-directional (upper) and directional (lower) probes showing magnetic flux and eddy current paths. (**b**) Equivalent circuit of probe and CFRP. Variation of output with FVF of (**c**) unidirectional and (**d**) cross-ply CFRPs, the former one gave a smooth calibration curve while the latter one with a certain amount of scatter. Reprinted with permission from Ref. [49] © 1976 Crown copyright.

Dingwall and Mead [49] studied the eddy current method to determine the FVF of CFRPs, with both non-directional and directional probes utilized (Figure 6a); the equivalent circuit of probe and CFRP is shown in Figure 6b, where a shunt resistance R*_s_* represents a possible transverse path due to fibres touching as an alternative to the capacitative path. Apparently, higher fiber volume fraction contributes to a higher number of conducting paths and greater response of the system [49]. Depth of penetration for both unidirectional and cross-ply CFRPs have been characterized and resulted with 8 mm and 1.8–2 mm respectively. The system was calibrated with different FVFs for both UD and cross-ply CFRPs via destructive acid burn-off method, in which the UD gave a smooth calibration curve (Figure 6c) while the cross-ply one with a certain amount of scatter (Figure 6d).

On the one hand, layer coupling alters eddy current paths, and transverse conductive paths of cross-ply CFRPs redirect magnetic fields, reducing probe response. On the other hand, eddy currents follow elliptical paths along fibers with transverse capacitive coupling for UD CFRPs, which can be modeled by coupled LCR circuits. Meanwhile, for cross-ply ones, conductive paths exist both within and between plies with unpredictability, and no valid model can be applied. As a consequence, the ECT method could be used to measure the FVF, which could be fairly accurate for UD CFRPs, but has limitations on the accuracy for cross-ply ones.

The method requires accurate calibration of the system, which needs calibrations for each probe, each resin system and the thickness of the sample. While there are difficulties in obtaining the specimens to produce such curves, the difficulties are not insuperable, and a viable system of measuring volume fraction seems possible [49]. After calibration, the FVF can be derived from

(20) $$V_{f}=f\left(V_{output},t\right)$$

where f is a lookup function, $V_{output}$ is the output voltage and t is the thickness of the sample.

## 4. Discussion and Conclusions

Although differences exist in the coefficient of thermal expansion (CTE) between reinforcing fibers and polymer matrices, particularly for natural fibers and thermoplastics exhibiting higher CTE values, this review does not address temperature effects on fiber volume fraction. Carbon, glass, and Kevlar fibers demonstrate CTEs ranging from 10^−6^ K^−1^ to 10^−5^ K^−1^, and natural fibers also below 10^−4^ K^−1^. Polymer matrix CTEs typically span 10^−6^ K^−1^ to 10^−4^ K^−1^. FRP composites exhibit bulk CTEs below 10^−4^ K^−1^, rendering thermal expansion negligible for fiber volume fraction measurements [55,56,57].

Traditional destructive methods, in particular combustion and dissolution methods, have been widely used with the standards developed, and also applied to calibrate the newly developed non-destructive methods, indicating their reliability, high accuracy and cost efficiency. Meanwhile, they are facing challenges of destructing samples, medium to high time consumption, as well as requiring fume extraction to deal with emitted toxic gases, hazardous chemicals and complex waste disposal. In addition, reinforcing fibers will also be attacked; left regions of undigested polymer also exist. To ensure a selective digestion of polymer matrix, a selective microwave-assisted sample preparation protocol has been developed and reported by National Physical Laboratory [58].

To overcome the challenges of destructive methods, and also fulfill the increased demands in non-destructive testing, various methods have been developed and summarized in Table 1.

Cross-sectional image analysis and X-ray CT method contribute to 2D and 3D visualization respectively, with the former one requires destructive polishing, and both of them are sensitive to the threshold selection which largely affects the testing accuracy. For the synchrotron X-ray CT imaging, the volume scanned is rather small compared to composite structures, while for X-ray CT imaging bigger components suffer from poor phase contrast and long acquisition times. The image contrast relies on differences in the attenuation of X-ray paths through the object under investigation [30]. Initial studies were performed on the images by manually choosing the threshold value which can be subjective. However, considering the sensitivity of the proposed methodology to the threshold value, choice of this value was later automated by choosing an appropriate algorithm [59]. In combination with modern scanners enabling multiscale analysis, high accuracy could be achieved using the X-ray CT method.

Thickness method presents significant advantages in terms of cost-effectiveness and procedural simplicity, while limited to flat panels with reinforcement of known and consistent areal weight.

As thermography is a non-contact method with the infrared camera used for detecting the temperature, it can be applied to large areas and complex geometries, e.g., curved surfaces; also, it is efficient and safe, with no harmful byproducts. However, it has limited depth sensitivity, and its applicability depends on materials; if the materials have low thermal emissivity, then it is difficult to measure. In addition, the camera is sensitive to a wide range of noises, affecting many pixels randomly or creating random patterns [60]. The field of pulsed thermography is developing methods to improve the detectability, in combination with signal and data processing [60,61,62].

For the ultrasonic method, effects of temperature and moisture on the material will influence ultrasonic wave propagation and velocity [63], which could be further investigated to achieve high-precision characterization. Moreover, ultrasound is very sensitive to porosity and is one NDT technique that can effectively detect delamination and debonding in CFRP materials, which are critical defects affecting the internal structure of composite materials. Combining the ultrasonic method to measure porosity with thermography for fiber volume fraction can contribute to higher accuracy.

Eddy current testing (ECT) method has the advantage of quicker inspection capability, lower cost and ease of use, which can be designed to be lightweight and portable for in-line monitoring. However, it requires accurate calibration for each probe, resin system and thickness, after which it could be used for conductive materials such as CFRPs with appropriate accuracy for unidirectional panels. Similarly, XRD also needs calibration and is only valid for unidirectional composites, but for both conductive and non-conductive ones, e.g., CFRPs and GFRPs.

## Figures and Tables

**Table 1:** Comparison of measurement methods for fiber volume fraction of fiber-reinforced polymer composites.

Methods		Fiber Volume Fraction	Cost	Time Consuming	Accuracy	Limitations
Destructive	Combustion	$V_{f}=\frac{M_{f}}{M_{c}}\times \frac{\rho_{c}}{\rho_{f}}$	Low	Medium	High	Combustibility/digestibility of the resin needs to be considered.
	Dissolution		Low	High	High	
	Cross-sectional image analysis	$V_{f}=\frac{\sum_{i=1}^{n}V_{fi}}{n}$ $,\;V_{fi}=\frac{N_{i}\cdot \bar{A_{f}}}{A_{i}}$	Low	Low	Medium	Fabric-reinforced composites are not available, and are sensitive to the threshold selection.
Non-destructive	Thickness	$V_{f}=\frac{nA_{w}}{\rho_{f}d}$	Low	Low	Medium	Limits to flat panels with reinforcement of known and consistent areal weight.
	X-ray CT	$V_{f}=\frac{\sum_{i=1}^{n}V_{fi}}{n}$ $,\;V_{fi}=\frac{N_{i}\cdot \bar{V_{f}}}{V_{i}}$	High	High	Medium to high	Trade-off between component scale and accuracy, also sensitive to the threshold selection.
	Thermography	$({\rho c)}_{c}=V_{f}({\rho c)}_{f}+(1-V_{f}-V_{v})({\rho c)}_{m}$	Medium	Low	Medium to high	Limited depth sensitivity and difficult to measure materials with low thermal emissivity.
	Ultrasound	$V_{f}=\frac{v_{mea}+v_{void}-v_{resin}}{v_{fibre}-v_{resin}}$	Medium	Low	Medium	Sensitive to temperature and moisture.
	XRD	$1/V_{f}\propto Peak\;height$	Medium	Low	Medium	Needs calibration and only valid for unidirectional composites.
	Eddy current	$V_{f}=f(V_{output},t)$	Medium	Low	Medium	Needs massive calibration and only valid for conductive materials, e.g., CFRPs, and only accurate for unidirectional panels.

## Data Availability

No new data were created or analyzed in this study. Data sharing is not applicable to this article.
